# Rare Case of Tacrolimus-Induced Acute Pancreatitis in a Liver Transplant Recipient

**DOI:** 10.14309/crj.0000000000001659

**Published:** 2025-04-25

**Authors:** Renan Prado, Arjun Chatterjee, Zurabi Zaalishvili, Rupayan Kundu, Shilpa Junna, Shreya Sengupta, Nizar Zein, Syed A. Mohiuddin

**Affiliations:** 1 Department of Internal Medicine, Cleveland Clinic Foundation, Cleveland, OH; 2 Department of Gastroenterology and Hepatology, Digestive Disease Institute, Cleveland Clinic, Cleveland, OH

**Keywords:** tacrolimus induced acute pancreatitis, drug induced acute pancreatitis, DIP, pancreattiis, tacrolimus

## Abstract

Tacrolimus-induced acute pancreatitis (TAP) is rare and requires thorough evaluation, including genetic testing, to rule out other causes. While TAP has been documented in a few cases following kidney transplantation, we report the first case of TAP in an adult after liver transplantation, with a noteworthy feature of late-onset TAP occurring more than 12 months after initiating tacrolimus therapy. This case highlights the potential for delayed onset of TAP, and we suggest that tacrolimus may warrant reclassification from category III to Ic in the drug-induced pancreatitis classification. In addition, we introduce sirolimus as a viable alternative for immunosuppression following TAP.

## INTRODUCTION

Tacrolimus-induced acute pancreatitis (TAP) is a rare condition that requires extensive workup with exclusion of other common causes of acute pancreatitis (AP), followed by modification of tacrolimus dose and goal tacrolimus trough level. We describe a rare case of TAP in a liver transplant patient.

## CASE REPORT

**Figure 1.**
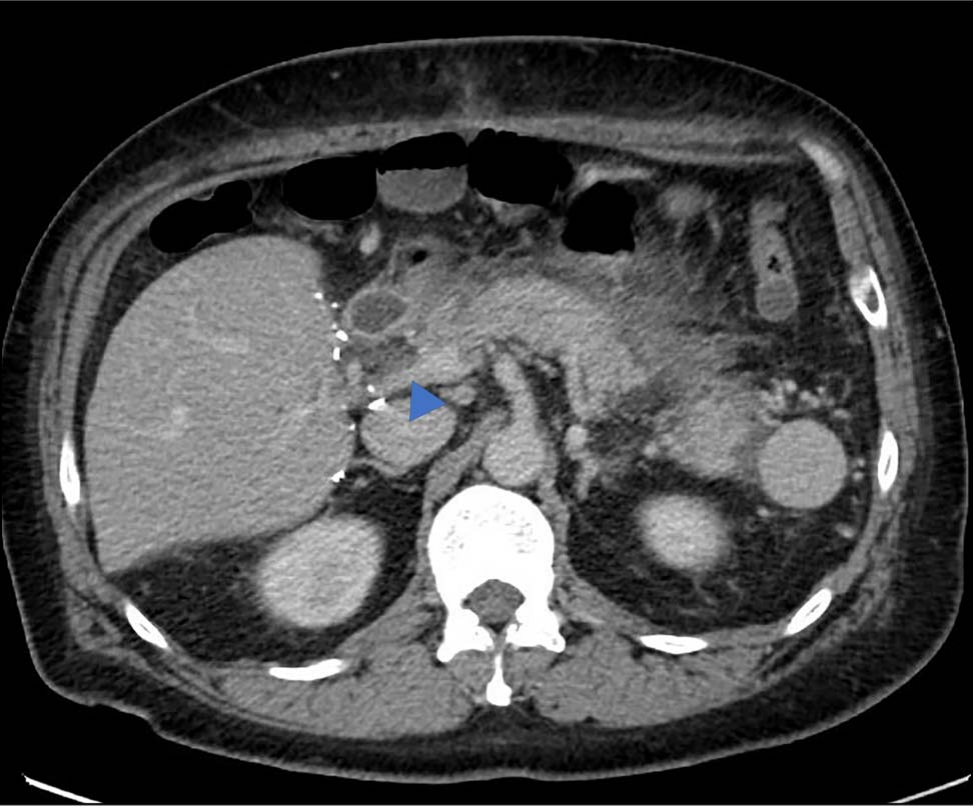
Abdominal computed tomography showing inflammation and fat stranding surrounding the pancreas and a small fluid collection of 2.0 × 1.2 cm in the pancreatic head (blue arrowhead).

**Figure 2.**
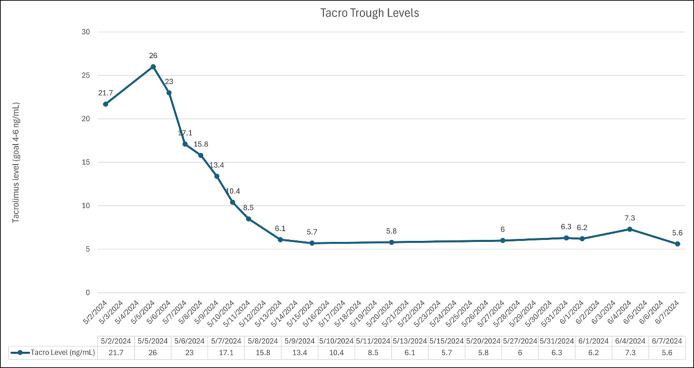
Tacrolimus trough levels during initial presentation and follow-up.

A 48-year-old man with a medical history of alcoholic cirrhosis, status postliver transplant 43 months ago, on tacrolimus since the surgery, cholecystectomy, and splenectomy presented with 3-day history of nausea and epigastric abdominal pain with radiation to the back. He denied fever, vomiting, hematemesis, melena/hematochezia, jaundice, pale stools, or prior episodes of AP. He is a nonsmoker and denied current alcohol use. Laboratory results on admission revealed leukocytosis, elevated lipase (677 U/L), and tacrolimus (21.7 ng/mL) levels (Table 1). Liver transaminases and bilirubin were normal. Computed tomography of the abdomen and pelvis performed demonstrated pancreatic inflammation and fat stranding consistent with AP. A pancreatic head pseudocyst (2.0 × 1.2 cm) and a new nonocclusive portal vein thrombus extending to superior mesenteric vein were also noted (Figure 1). Upper endoscopy with endoscopic ultrasound was unremarkable for microlithiasis or occult lesions. Other causes of AP, including hypertriglyceridemia, IgG4-related disease, cytomegalovirus, and ethanol intake (with multiple negative levels of phosphatidylethanol) were excluded. Genetic panel for AP was negative (Table 2). Based on an elevated tacrolimus trough level of 26 ng/mL, the most probable cause was determined to be TAP. The moderately severe AP was treated with fluid resuscitation, and tacrolimus dosage was reduced with weekly monitoring targeting a goal trough 4–6 ng/mL (Figure 2).

**Table 1.** Laboratory values on admission

Test	Value	Normal range
White blood cells	13.31 k/µL	3.70–11 k/µL
Red blood cells	4.27 m/µL	4.2–6 m/µL
Platelets	487 k/µL	150–400 k/µL
Total protein	6.8 g/dL	6.3–8 g/dL
Albumin	2.6 g/dL	3.9–4.9 g/dL
Total calcium	8.3 mg/dL	8.5–10.2 mg/dL
Total bilirubin	1.4 mg/dL	0.2–1.3 mg/dL
Alkaline phosphatase	315 U/L	38–113 U/L
Aspartate aminotransferase	89 U/L	14–40 U/L
Alanine aminotransferase	72 U/L	10–54 U/L
Glucose	106 mg/dL	74–99 mg/dL
Blood urea nitrogen	21 mg/dL	9–24 mg/dL
Creatinine	0.67 mg/dL	0.73–1.22 mg/dL
Sodium	136 mmol/L	136–144 mmol/L
Potassium	4.7 mmol/L	3.7–5.1 mmol/L
Chloride	104 mmol/L	97–105 mmol/L
Bicarbonate	24 mmol/L	22–30 mmol/L
Anion Gap	8 mmol/L	9–18 mmol/L
Lipase	677 U/L	16–61 U/L
GGT	270 U/L	10–70 U/L
Triglycerides	84 mg/dL	<150 mg/dL
IgG4	42 mg/dL	3.9–86.4 mg/dL
Cytomegalovirus DNA	Not detected	N/A
Phosphatidylethanol	<10	<10: Not detected

GGT, Gamma-glutamyl transferase.

**Table 2.** SNaP-SHOT pancreatitis final report (detailed genetic findings) from saliva DNA

Groups	Gene mutations	Association with
Pancreatic acinar cell risk	*CLDN2*	Increased risk to progress from AP to CP
Pancreatic acinar cell risk	*PRSS1*	Increased risk to progress from AP to CP; increase severity of AP
Pancreatic duct cell risk	*CASR*	May be associated with an increase odd of pancreatitis when associated with some genes mutations, such as *CTRC, CFTR*, and *SPINK1*
Hyperglyceridemia	*APOA5*	Increase risk of hypertriglyceridemia
Hyperglyceridemia	*APOB*	Increase risk of hypertriglyceridemia

No mutations in the genes: *ABCG8, CFTR, CTRC, GGT1, LDL, SLC10A2, SLC26A9*, and *SPINK1 *(None of the mutation is capable to increase the risk of AP).

AP, acute pancreatitis; CP, chronic pancreatitis.

He was seen in clinic 1 month after discharge and reported overall improvement in his symptoms, although he still experienced mild epigastric pain. Two months later, magnetic resonance imaging of the pancreas with contrast showed persistent signs of pancreatitis, including multiple peripancreatic and increasing intrapancreatic collections up to 3.8 cm, as well as a resolving nonocclusive thrombus in the superior mesenteric vein. He was readmitted with abdominal pain and lipase levels of 195 U/L for another episode of pancreatitis 10 weeks after the initial episode. At this point, tacrolimus was discontinued and sirolimus was initiated. He was treated with intravenous fluid resuscitation and a short course of opiates for pain relief. At a follow-up visit 1 week after discharge, he reported improvement in his symptoms with the use of Pancrelipase and switching immunosuppression to sirolimus.

## DISCUSSION

According to the American College of Gastroenterology, AP is defined by the presence of at least 2 of the following 3 criteria: characteristic abdominal pain, biochemical evidence of pancreatitis, and radiographic evidence of pancreatitis.^1^ AP is a well-recognized complication of organ transplantation, with an incidence of 1.5%–8% in liver transplant recipients.^2^ The use of various immunosuppressive agents, including corticosteroids, tacrolimus, cyclosporine, azathioprine, and mycophenolate mofetil, has been linked to the development of AP in post-transplant patients.^2^

Drug-induced pancreatitis (DIP) accounts for up to 5% of all AP cases and is recognized as the third most common cause of AP, after excluding alcohol use and gallstones.^3^ Historically, tacrolimus-induced AP is rare and has been considered a Class IV medication based on the Badalov classification system; however, a recent review categorized tacrolimus as a DIP Class III, even before some additional cases were reported.^4–6^

TAP was initially reported in 2 pediatric cases following liver transplants.^7,8^ In 2000, the first adult case was documented in a patient receiving tacrolimus for graft-versus-host disease prevention.^9^ This was followed by 4 additional case reports, which are summarized in Table 3.^10–13^

**Table 3.** Drug-induced pancreatitis classification by Wolfe et al^5^

Drug class	Definition
Class Ia	• At least 1 case report in humans, with positive rechallenge
	• All other causes, such as alcohol, hypertriglyceridemia (and hyperlipidemia), gallstones, and other drugs, are ruled out
Class Ib	• At least 1 case report in humans, with positive rechallenge
	• Other causes, such as alcohol, hypertriglyceridemia, gallstones, and other drugs, were not ruled out
Class Ic	• At least 1 case report in humans, without a positive rechallenge (ie, no rechallenge or a negative rechallenge) • Other causes, such as alcohol, hypertriglyceridemia, gallstones, and other drugs, are ruled out
Class II	• At least 2 cases in humans reported in the literature, without a positive rechallenge (ie, no rechallenge or a negative rechallenge)
	• Other causes, such as alcohol, hypertriglyceridemia, gallstones, and other drugs, were not ruled out
	• Consistent latency^a^
Class III	• At least 2 cases in humans reported the literature, without a positive rechallenge (ie, no rechallenge or a negative rechallenge)
	• Other causes, such as alcohol, hypertriglyceridemia, gallstones, and other drugs, were not ruled out
	• Inconsistent latency^a^
Class IV	• At least 1 case in humans reported the literature
	• Drugs not fitting into the earlier-described classes

a “Consistent latency” defined as >75% of cases falling into the same latency category. Category 1: <24 h, Category 2: 1–30 d, Category 3: >30 d.

Mallory and Kern established criteria to assess the likelihood of DIP. Their criteria include the following: (i) onset of pancreatitis during treatment with the suspected drug, (ii) resolution of pancreatitis after discontinuing the drug, (iii) exclusion of other possible causes, and (iv) recurrence of pancreatitis on re-exposure to the drug.^14^ In our case, the first 3 criteria are met. In addition, the onset of pancreatitis corresponded with high trough level of tacrolimus.

Our case presents several unique and noteworthy aspects. Although rare, tacrolimus-induced recurrent AP can occur and may be overlooked in the standard workup for pancreatitis. Although immunosuppression medication-associated AP is well known, to our knowledge, this is the only documented case of TAP where we were able to definitively rule out all other etiologies of AP, including biliary pancreatitis, alcohol abuse (confirmed by a negative phosphatidylethanol test), hypertriglyceridemia, hypercalcemia, viral infections, and genetic predispositions (negative SNaP-SHOT analysis for AP gene mutations). We also highlight the prolonged time between medication use and AP, and the use of sirolimus as an alternative after the TAP (Table 4). With current evidence and based on the DIP classification proposed by Wolfe et al, TAP should be reclassified as Badalov Class Ic.^6^ Physicians should consider tacrolimus a cause of AP postliver transplant and switch to other immunosuppressants such as sirolimus if indicated. Tacrolimus is primarily associated with nephrotoxicity, neurotoxicity, and new-onset diabetes, whereas sirolimus is more commonly linked to hyperlipidemia, impaired wound healing, and hematologic abnormalities. Both drugs elevate the risk of infections.^15,16^

**Table 4.** Previous case reports of drug-induced pancreatitis by tacrolimus in adult population

Author	Publication year	Country	Age/Sex	Tac levels	Indicatives	Diagnosis time (post-treatment)	Substitutive drug of choice
Nieto et al.^8^	2000	the United States	28/Female	17.6 ng/mL	Graft-versus-host disease	15 d	Held tacrolimus. No new drug
Im et al.^9^	2013	South Korea	22/Female	>30 ng/mL	Heart transplant	7 mo	Lower dose of tacrolimus
Xu et al.^10^	2019	China	45/Male	>30 ng/mL	Kidney transplant	20 d	Cyclosporine
Liu et al.^11^	2021	China	24/Male	>30 ng/mL	Kidney transplant	10 d	Cyclosporine
Ding et al.^12^	2022	China	38/Female	>30 ng/mL	Kidney transplant	68 d	Cyclosporine
This study	2024	the United States	49/Male	26 ng/mL	Liver transplant	43 mo	Lower dose of tacrolimus than sirolimus

In conclusion, although there is no consensus or guidelines about the tacrolimus trough level that should prompt evaluation for pancreatitis in liver transplant patients with epigastric pain, the presence of high levels of tacrolimus should create a suspicious of AP. Improving awareness of the condition and further reporting to enrich literature will help improve outcomes in patients with idiopathic pancreatitis taking immunosuppressive drugs such as tacrolimus.

## DISCLOSURES

Author contributions: A. Chatterjee, R. Prado, Z. Zaalishvili: writing the initial draft of the manuscript; S. Junna, S. Sengupta, N. Zein: drafting and critical review the manuscript; SA Mohiuddin: final approval of the manuscript. A. Chatterjee is the article guarantor.

Financial disclosure: None to report.

Informed consent was obtained for this case report.
